# What is the Origin of the Ectopic Beat?

**DOI:** 10.1007/s12471-015-0655-z

**Published:** 2015-02-11

## Rhythm puzzle

A 79-year-old man visited the outpatient clinic because of precordial pain and dyspnoea during mild exercise. These symptoms started 4 weeks ago. His physical condition, so far, had been excellent and this was the first time in his life that the family doctor had referred him to a cardiologist. As medication, he used simvastatin 20 mg once daily. Physical examination revealed no abnormalities: height 1.86 m, weight 71 kg, blood pressure 120/70 mm Hg, regular pulse of 60 bpm and during auscultation of the heart he had normal heart sounds. Laboratory investigation was unremarkable: C-reactive protein> 1, haemoglobin 8.0 mmol/l, creatinine 73 µmol/l, troponin I negative, glucose 5.8 mmol/l, total cholesterol 3.0 mmol/l and LDL cholesterol 2.1 mmol/l.

The ECG showed a sinus rhythm of 60 bpm with a normal PR interval, a prolonged QRS complex, around 120 ms with a left axis deviation compatible with a left anterior fascicular block according to the classical criteria (qR in aVL, rS in II, III, aVF) in the standard ECG leads. In leads II, III, aVF, and V4-V6 the repolarisation pattern was abnormal, showing asymmetrical negative T waves.

**Fig. 1:**
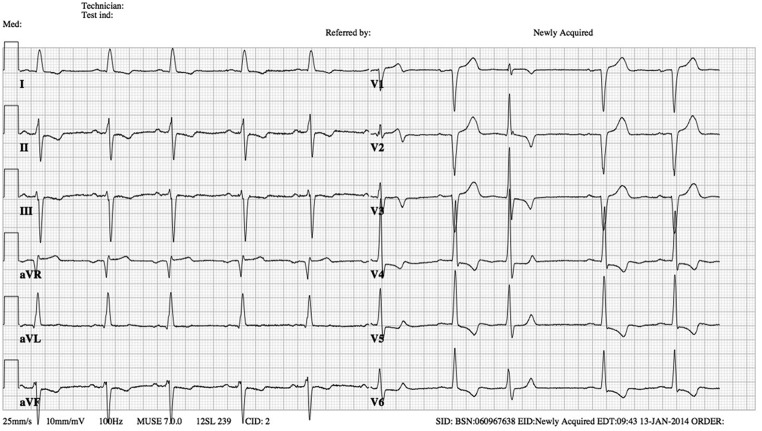
Twelve-lead ECG. What is the origin of the third beat in leads V1–V6?

In the precordial leads, there is a premature depolarisation following the second QRS complex and the question is what is the origin of this QRS complex and why does the QRS complex following the first sinus beat in V1–V6 have a different QRS morphology than the other QRS complexes during sinus rhythm (Fig. 1)?

## Answer

You will find the answer elsewhere in this issue.

